# Prevalence of malnutrition in pediatric pulmonary hypertension cohort and role for registered dietitian involvement

**DOI:** 10.3389/fped.2023.995470

**Published:** 2023-04-04

**Authors:** Presley R. Crowell, Mackenzie R. Frederick, Rozmeen A. Fombin, Nidhy P. Varghese, Fadel E. Ruiz

**Affiliations:** ^1^ Department of Clinical Nutrition, Texas Children’s Hospital, Houston, TX, United States; ^2^ Department of Pulmonary Medicine, Texas Children’s Hospital, Houston, TX, United States; ^3^ Department of Pediatric Pulmonology, Baylor College of Medicine, Texas Children’s Hospital, Houston, TX, United States

**Keywords:** malnutrition, registered dietitian, pediatric nurition, nutrition assessment, pulmonary hypertension

## Abstract

**Introduction:**

Pediatric pulmonary hypertension (PH) is a serious condition with increased risk for malnutrition due to increased caloric needs and reduced energy intake. This combination of disease and dynamic elements make it particularly challenging to meet expected growth patterns. Pediatric PH patients require close monitoring and individualized nutrition interventions to best meet nutrient needs. The prevalence of malnutrition and effective nutrition interventions in pediatric PH has not been studied.

**Methods:**

Using our electronic medical record (EMR) patient care dashboard, malnutrition prevalence was assessed by reviewing the active problem list of all active PH patients at our center. A chart review compared patients with diagnosed malnutrition in the EMR to those with malnutrition identified by a registered dietitian (RD) using a standardized tool. Chart reviews also assessed outcomes of RD interventions.

**Results:**

195 patients were identified as active PH patients followed by our PH center during the study period (November 2021 to January 2023). Of these, 5% (10/195) had an ICD-10 code for malnutrition listed in their chart. However, upon further chart review of the remaining 185 patients, 22% (41/185) had malnutrition identified by a RD using Texas Children's Malnutrition Tool, totaling 51/195 (26%) malnourished patients. The PH RD saw 25/51 (49%) patients during PH clinic visits in the study period. At follow up visits (3–4 months after initial assessment), 56% (14/25) patients seen by the PH RD either improved or resolved their malnutrition status by *z*-score assessment.

**Conclusion:**

Malnutrition is present in pediatric PH, although underappreciated and underdiagnosed. Managing malnutrition in pediatric PH requires close monitoring, multidisciplinary involvement, and individualized nutrition recommendations. This is best achieved by a dedicated PH RD who is familiar with the unique needs of this population and available to provide consistent nutritional assessments and interventions to reduce malnutrition in this population.

## Introduction

Pediatric malnutrition is defined as an imbalance between nutrient requirements and nutrient intake that results in deficits of energy, protein, or micronutrients that may negatively affect growth, development, and long-term health outcomes (1, 2). Even with the knowledge of increased consequences of pediatric malnutrition, pediatric populations continue to suffer from undernutrition. This condition is often underreported, and underappreciated. Prior studies have found malnutrition was only coded in 4% of hospitalized pediatric patients, despite reports of rates of 24%–50% of pediatric hospitalized patients (2).

Patients with chronic disease are at an increased risk for developing malnutrition due to increased energy expenditure. Malnutrition in the setting of chronic illnesses can be multifactorial, requiring complex individualized nutrition interventions. In addition, poor nutritional status can be an independent predictor of morbidity and mortality (3). A study by Luo and associates found that in adult pulmonary hypertension (PH) patients, poor nutritional status was associated with increased risk of adverse outcomes, including death. Worsening nutrition status was also linked to poorer lung function on pulmonary function tests (4).

Chronic lung disease in general increases energy expenditure and energy needs for growth in children; the same is true for those with cardiovascular disease. Therefore, pediatric PH has increased risks of malnutrition in an already vulnerable patient population. Limited research has been done to examine the energy requirements of adults with PH, and even less research is available on energy requirements for pediatric PH patients (5). One study conducted by Blasquez and associates examined nutrition status of children under the age of 2 with congenital heart disease (CHD). This study found moderate to severe malnutrition was significantly more common in children with PH. This study also found that those with PH had limited energy intake compared to those with CHD alone and those with PH had inadequate nutrition support (6). The combination of increased energy needs coupled with decreased intake puts pediatric PH patients at elevated risk for poor growth, chronic malnutrition, and negative health outcomes (7). For these reasons, pediatric PH patients are a unique group of children who require close monitoring and individualized nutrition interventions to meet growth expectations. Unfortunately, there is currently no validated screening process or standardized nutrition recommendations to address these specific nutrition concerns related to pediatric PH.

We therefore aimed to understand the prevalence of physician-diagnosed malnutrition in our PH cohort, compare this to rates of malnutrition identified by standardized assessment and examine nutritional outcomes of PH-dedicated Registered Dietitian (RD) assessment and intervention. We expected that increased RD involvement by a designated PH RD would improve weight gain, body mass index (BMI)/weight-for-length *z*-scores and overall nutrition status.

## Methods

This was a single center, retrospective cohort study of pediatric patients with diagnosed PH from November 2021 to January 2023. The study population included all active patients with PH managed at our PH center during the study period. Identified charts were assessed for ICD-10 codes for malnutrition (ICD 10- codes E43, E44, E46), which were listed in the active problem list of the EMR. Texas Children's dietitians applied the Texas Children's Pediatric Malnutrition Tool (Table 1) to the remaining active patient charts without ICD-10 code of malnutrition for missed malnutrition diagnoses. Analysis compared rates of malnutrition diagnosed and listed in the EMR to malnutrition identified by a RD. RD consults and nutrition interventions were assessed from November 2021 to January 2023 for impacts of RD involvement in the outpatient setting on malnutrition status.

**Table 1:** Patient characteristics.

	Active PH patient cohort	Malnourished PH patients	Mild malnutrition	Moderate malnutrition	Severe malnutrition
Age					
0–2 year	33	11	6	2	3
3–6 years	62	17	9	3	5
7–10 years	41	11	6	3	2
11–15 years	41	9	5	3	1
16–18 + years	18	3	1	2	0
Gestational age					
22–28 weeks	24	6	5	1	0
29–32 weeks	16	5	0	2	3
33–37 weeks	59	17	12	4	1
38 + weeks	91	21	10	4	7
Unknown	5	2	0	2	0
Provider documented PH severity					
Mild	67	12	7	4	1
Moderate	62	16	12	1	3
Severe	40	14	4	5	5
Resolved	16	6	4	1	1
Unknown/undocumented Severity	10	3	0	2	1
NYHA functional class					
1	71	14	10	4	0
2	69	19	9	6	4
3	14	4	2	1	1
4	4	3	1	1	1
Undocumented	37	11	5	1	5
Gastrostomy tube in place					
Yes	76	26	11	7	7
No	119	25	16	6	4
Comorbidities as per medical problem list					
Bronchopulmonary dysplasia	26	7	5	1	1
Congenital heart disease^a^	168	26	12	7	7
Chronic lung disease	15	15	5	4	6
Obstructive sleep apnea	17	3	1	0	2
Trisomy 21	32	3	3	0	0
Restrictive lung disease	4	0	-	-	-
Congenital diaphragmatic hernia	19	12	7	2	3
Other genetic diseases, known syndromes or associations^b^	28	12	6	5	1

^a^ Congenital heart disease: hypoplastic left heart syndrome, atrial septal defect, ventricular septal defect, atrioventricular septal defect, patent ductus arteriosus, patent foramen ovale, pulmonary vein stenosis, Scimitar syndrome, single ventricle physiology.

^b^ Genetic diseases or known syndromes/associations: Noonan syndrome, DiGeorge syndrome, Wolff Parkinson White syndrome, Ehlers-Danlos syndrome, Cantú syndrome, autoimmune disease not otherwise specified, cerebral palsy, Trisomy 18, Scimitar chromosomal duplication not otherwise specified, Adams Oliver syndrome, tetrasomy 9p, hereditary hemorrhagic telangiectasia gene mutations, Jeune syndrome.

## Results

**Figure 1:**
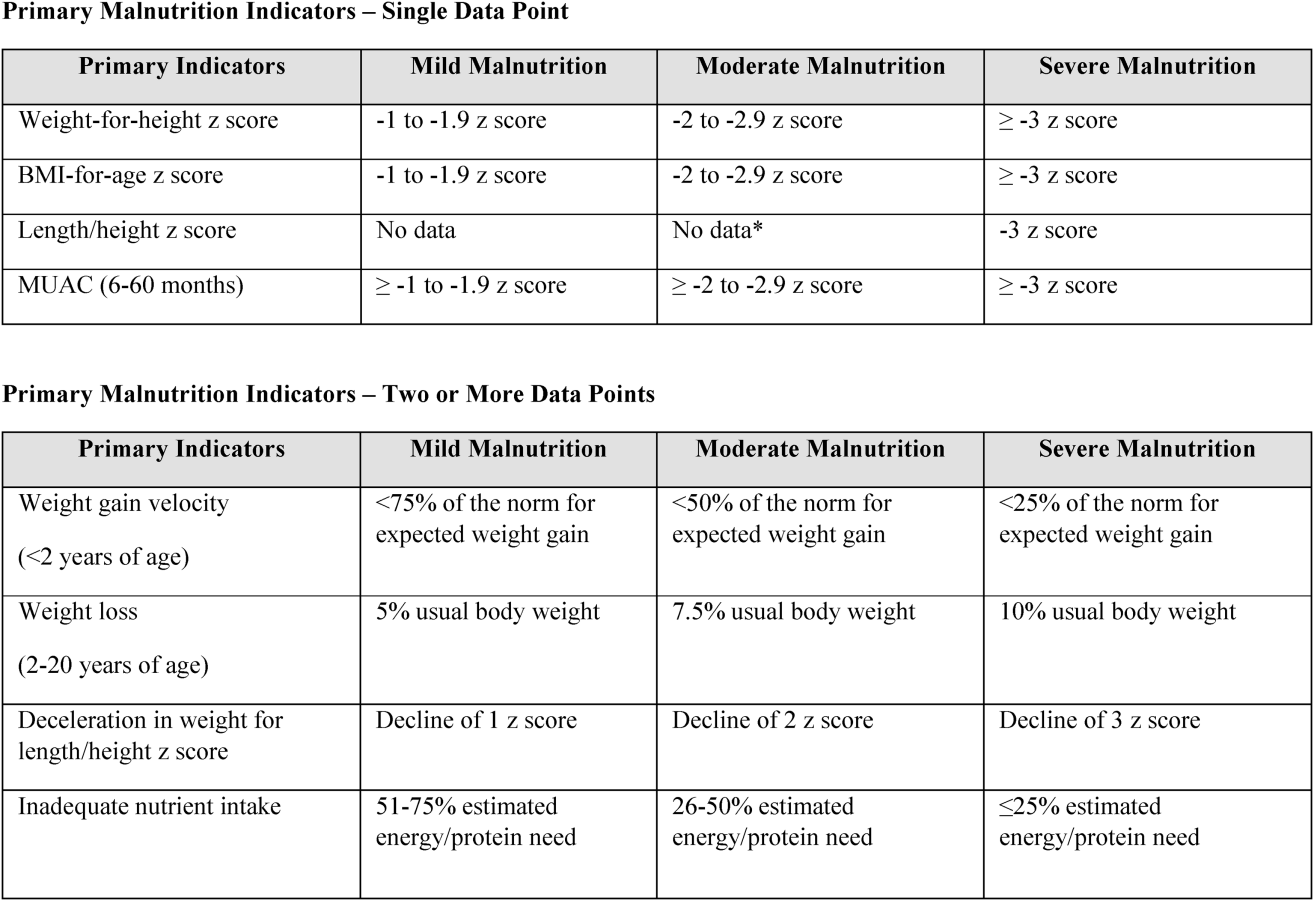
Texas children's hospital pediatric malnutrition tool (8).

195 patients were active during the study period (Table 1). Five percent (10/195) of patients at our PH center had an ICD-10 code for malnutrition in the EMR. Additional manual chart review of remaining 185 active PH patients identified an additional 41 (41/185, 22%) patients who met malnutrition criteria using the Texas Children's Hospital Pediatric Malnutrition Tool (Figure 1).

Of the 51 total patients identified with malnutrition, 27 patients (53%) were classified as mild malnutrition, 13 patients (25%) as moderate malnutrition, and 11 patients (22%) with severe malnutrition based on stratification of body mass index (BMI)/weight-for-length *z*-scores. Of note, severity of malnutrition approximated severity of PH. Table 1 shows malnutrition tended to occur more commonly in patients with moderate to severe PH. Severe malnutrition was more commonly seen in patients with New York Heart Association (NYHA) functional class ≥2, and mild malnutrition appeared more common in those with NYHA functional class 1. Of note the malnourished patient population was found to have higher prevalence of other comorbidities compared to the total PH patient population. Statistical analysis of the association between severity of PH and malnutrition status could not be performed due to sample size limitations (see Table 1).

**Figure 2:**
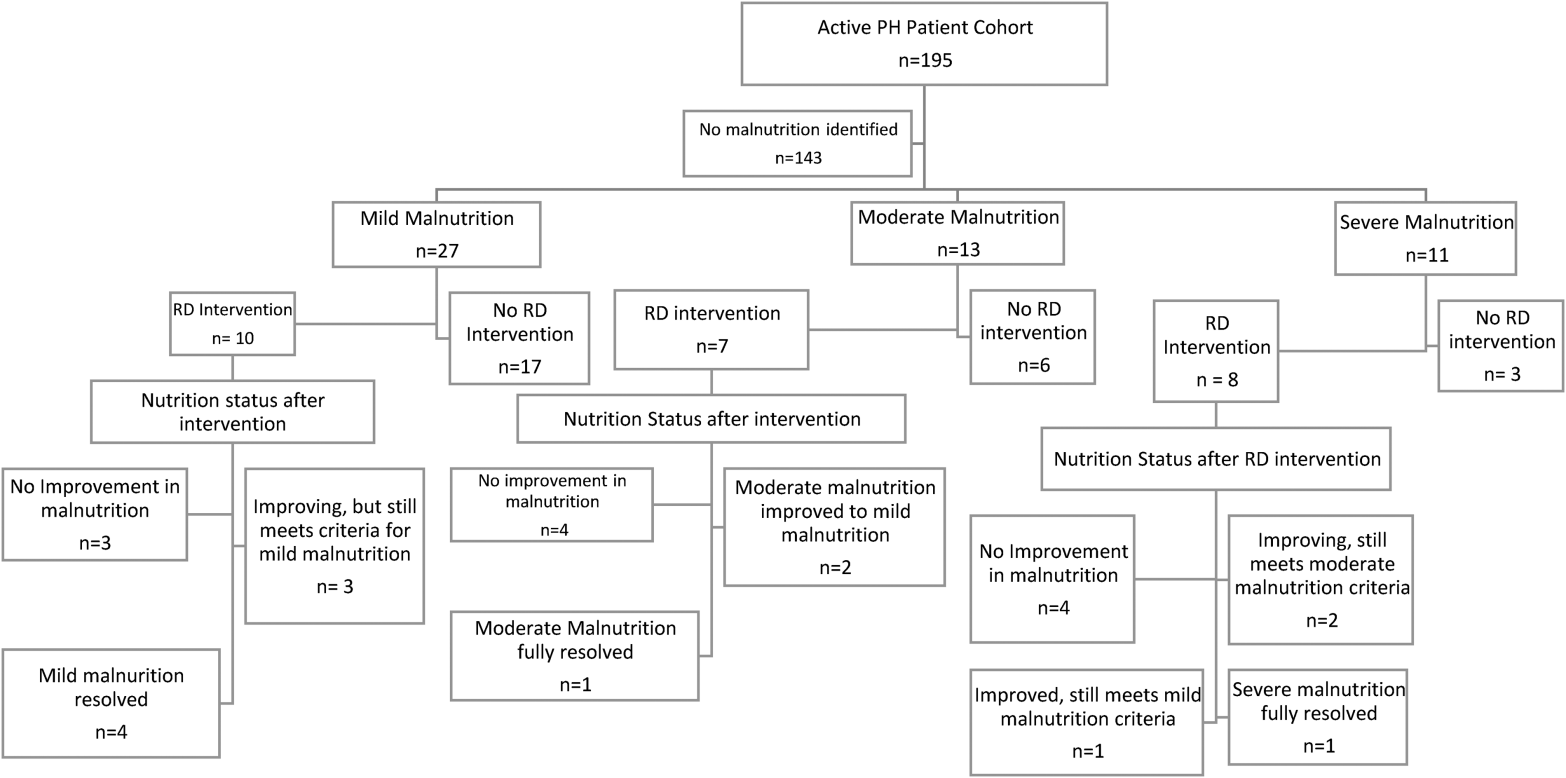
Flowchart of results.

Twenty-five of the 51 malnourished patients were seen by the PH RD for nutrition assessments. The remaining 26 patients were not seen due to RD coverage, inadequate time in clinic, virtual visit types or physician failure to consult RD. The designated PH RD screened clinics and reassessed malnourished patients typically every 3–4 months until patients met growth expectations and/or malnutrition status improved. Nutrition assessments included initial RD assessment of caloric intake and growth patterns. Fourteen of the 25 (56%) patients who had RD involvement either improved or resolved their malnutrition status based on improvement in BMI *z*-score at follow up PH clinic visits during the study period (see Figure 2).

## Discussion

PH has many risk factors for abnormal nutrition status. Dyspnea is a common symptom of PH. In addition to increasing energy needs, oral intake with dyspnea may be limited due to poor coordination of breathing and swallowing, with associated risk of dysphagia and aspiration. This increased effort can prevent adequate calorie consumption and may require the use of enteral nutrition support to safely meet elevated energy needs. Additionally, prolonged hospital stays decrease the ability to practice age-appropriate feeding skills and increases the incidence of oral aversions, which can limit age-appropriate foods consumed and further decrease caloric intake (9). Use of nutritional supplements or enteral nutrition support may therefore also be needed to meet calorie and protein needs in patients with oral aversions. Finally, medications used to manage PH symptoms may cause nausea, diarrhea, decreased appetite, and further decrease oral intake. The combination of increased energy needs coupled with decreased intake puts pediatric PH patients at elevated risk for poor growth, chronic malnutrition, and negative health outcomes due to decreased vitality (7). For these reasons, pediatric PH patients are a unique group of children who require close monitoring and individualized nutrition interventions to meet growth expectations.

In our cohort, only 5% of patients had a diagnosis of malnutrition listed in the EMR. However, manual chart reviews found malnutrition was prevalent at much higher rates of 26% (51/195) using a dedicated nutritional screening tool. The Texas Children's Malnutrition Tool is a standardized tool that is used throughout the institution to screen and assess for malnutrition (8). Per hospital policy, it is required for RDs to document using Texas Children's Pediatric Malnutrition tool upon identification of malnutrition. RDs receive extensive training on how to use the Texas Children's Pediatric Malnutrition Tool in multiple hospital settings to accurately identify malnutrition. RD findings of malnutrition are documented within provider encounters.

Our study found that malnutrition was commonly not coded as a medical problem, resulting in decreased team awareness of malnutrition, missed opportunities for RD consultation and fewer occasions for interventions to address malnutrition. At our institution, physicians are responsible for adding and identifying appropriate malnutrition ICD-10 codes. However, with an embedded RD in clinic and commitment for yearly nutritional screening, collaboration with a PH RD will improve accurate identification of malnutrition and promote essential communication among the medical team. This is essential for improved EMR capture of the malnutrition burden of nutritional concerns in this population and allow for appropriate interventions to reduce severity. Prior to the study period, RD assessments were on a consultation basis only. These were most often for poor weight gain, weight loss, decreased intake, or adjustments of enteral nutrition support. Dietitian interventions for these consults included introducing nutrition supplements, modifying enteral nutrition feeding regimens, and educating families on high calorie foods. In addition to assessments, RDs assisted providers in ordering nutrition supplements and specialty enteral formulas to best meet patient needs. Advocating and facilitating access for patients to receive necessary supplies to meet their increased energy needs was a recognized crucial component of RD involvement to improve nutrition outcomes. Unfortunately, during this period, patients were seen on a consult-only basis and there was no structured RD follow up nor tracking of consultation. On average, staff RD were consulted on 4–6 patients per month.

In November 2021, our PH center acquired a designated PH RD to have embedded support within the clinic, to increase access to consultation and intervention. The PH RD communicated with families in between PH clinic appointments on a bi-weekly to monthly basis to ensure growth patterns were following age-appropriate recommendations. Oral nutrition supplements and enteral nutrition were assessed and adjusted as indicated to meet constantly fluctuating energy needs due to changes in activity, intake, or acute illnesses. Nutrition reassessments were conducted as determined by the PH RD if growth remained below recommended for age, suboptimal intake continued, or malnutrition persisted. On average, the PH RD consults on 25–30 new and follow up patients per month. RD re-assessments in PH clinic are conducted 3–4 months after the initial RD assessment.

Active interventions, close monitoring and frequent follow up by a designated PH dietitian improved or resolved malnutrition status in 56% of patients followed by the PH RD from November 2021 to January 2023. Our findings supported our expectation that increased RD involvement by a designated PH RD would be associated with improved weight gain, BMI/weight-for-length z scores and overall nutrition status.

Limitations of this study include single center study with a limited sample size. Inherent to retrospective chart review analyses, there may have been missed malnutrition diagnoses by providers and missed malnutrition documentation in the absence of RD consultation. We did not have year-over-year data to offer direct comparisons prior to November 2021, although this information is now available for future tracking. Although nutritional outcomes were improved, the study period was too short to identify other nutrition-related improvements on objective markers of PH disease severity such as hemodynamics. NYHA FC was not significantly affected by nutritional intervention since most children are FC I–II, consistent with registry data (10).

## Conclusion

Nutritional assessment is a crucial component of comprehensive pediatric PH care. The vulnerable nature of pediatric PH requires close assessment and monitoring of energy expenditure and calorie supplementation by a trained dietitian who is familiar with nutritional requirements of children with cardiopulmonary disease. Our study found that a designated PH RD increased awareness of malnutrition and improved the nutrition status of many patients seen during a designated period at our PH center. The RD was able to identify patients at risk of developing malnutrition using a standardized screening tool and recommend appropriate nutrition interventions, which resulted in improved nutritional outcomes. Further studies to provide guidelines and dietary recommendations for this population are needed.

## Data Availability

The raw data supporting the conclusions of this article will be made available by the authors, without undue reservation.
